# Lifestyle factors and psychological factors are associated with central pain processing in service members with persistent low-back pain: A cross-sectional exploratory study

**DOI:** 10.1097/MD.0000000000036741

**Published:** 2023-12-22

**Authors:** Julia M. Prent, Peter van der Wurff, Gwendolyne G.M. Scholten-Peeters

**Affiliations:** a Department of Human Movement Sciences, Faculty of Behavioural and Movement Sciences, Vrije Universiteit Amsterdam, Amsterdam Movement Sciences Program Musculoskeletal Health, Amsterdam, The Netherlands; b Research and Development, Military Rehabilitation Centre “Aardenburg”, Doorn, The Netherlands.

**Keywords:** central nervous system sensitization, endogenous analgesia, military personnel, musculoskeletal pain, quantitative sensory testing

## Abstract

Persistent low-back pain (LBP) is highly prevalent in the military. Altered central pain processing is one of the mechanisms found to underlie persistent LBP. Our aim was to explore which factors are associated with altered pain processing in Dutch service members with persistent LBP. This knowledge may guide clinicians in what factors to address in the treatment of dysfunctional pain processing in service members with persistent LBP. Twenty-one service members with persistent LBP (mean age 34.0 years, 18 males) were included in this cross-sectional exploratory study. Participants completed questionnaires regarding lifestyle and psychological factors. Altered central pain processing was measured by temporal summation of pain to examine the function of the pain facilitatory system and by conditioned pain modulation to examine the pain inhibitory function. Univariable and multivariable linear regression analyses were performed. A higher local temporal summation of pain was associated with a longer sitting time, a higher level of physical activity and a higher level of pain catastrophizing. A higher local conditioned pain modulation was associated with a higher level of pain catastrophizing, anxiety and depression symptoms, and with a lower sleep quality. A higher remote conditioned pain modulation effect was associated with a higher level of physical activity, a higher body mass index and a shorter sitting time. This study succeeded in identifying lifestyle and psychological factors associated with altered pain processing in service members with persistent LBP. Prospective studies are needed to examine causality in these relationships.

## 1. Introduction

Persistent low-back pain (LBP) is one of the most prevalent pain conditions in the military.^[1,2]^ Consequences of persistent LBP among service members include reduced odds of returning to duty and a high prevalence of opioid use in the United States.^[3,4]^ These findings underpin the importance of improving the management of LBP in the military.^[5]^

The current difficulty in treating persistent LBP is that 85% of the patients are classified as having nonspecific LBP, meaning there is no detected pathoanatomical origin of the pain.^[6,7]^ Consequently, management merely focuses on reducing pain and disability, without considering the underlying processes of pain.^[8]^ Though this approach has been shown to be effective to some extent, effect-sizes of such treatments are generally small.^[9]^ Numerous researchers advocate that the effectiveness of pain treatments can be improved by identifying and specifically targeting the actual biological mechanisms underlying the pain.^[8,10–12]^

One of the proposed mechanisms underlying persistent LBP is altered central pain processing.^[13]^ Dysfunctional pain processing may induce an enhanced nociceptive transmission and therefore elicit pain hypersensitivity.^[14]^ Two systems are found to play an important role in central pain processing; the ascending facilitatory- and the descending inhibitory system.^[13,15]^ The facilitatory function of the ascending system can be enhanced through an increased excitability of spinal wide dynamic range neurons in the dorsal horn.^[13,16]^ The descending system can be limited in the capacity to inhibit pain through a reduced functioning of endogenous analgesic processes.^[13,17]^

To identify altered pain processing in LBP patients, the functioning of the pain facilitatory and inhibitory system should be quantified.^[18,19]^ Since direct measurements of these neurophysiological mechanisms are not possible in humans, derivatives of these processes are measured through quantitative sensory testing (QST).^[20]^ The function of the pain facilitatory system is estimated by the temporal summation of pain (TS) test. TS measures the increase in pain, through spinal excitation, as a response to repetitive noxious stimuli of a constant intensity within the same area.^[16,21,22]^ The pain inhibitory function is estimated using the conditioned pain modulation (CPM) test. CPM is based on a “pain inhibits pain” model and measures to what extent a noxious stimulus at one body part reduces pain, through the activation of descending inhibitory pathways, initiated by a noxious stimulus at another body part.^[17,23,24]^ TS and CPM have been used in previous studies to assess the role of altered central pain processing in LBP, by comparing the pain inhibitory and facilitatory function between LBP patients and healthy controls.^[21]^ Recent systematic reviews show a tendency towards an enhanced TS and reduced CPM in LBP patients compared with healthy controls.^[22–24]^ However, the results are inconsistent and reflect inter-individual differences in pain processing.^[25]^ This variance in pain processing was found to be associated with lifestyle factors including age, alcohol consumption, sleep quality, physical activity and with various psychological factors.^[26,27]^

Although previous studies succeeded to identify some factors associated with altered pain processing, these factors remain unknown in service members with LBP, and have not been assessed so far. Considering the high level of physical fitness demanded for military service,^[28]^ differences in health and health behavior between service members and civilians,^[29]^ and the presence of particular psychological problems in service members,^[30]^ it is of importance to explore these factors in this target population. This knowledge may guide clinicians in what factors to address in the treatment of dysfunctional pain processing in service members with persistent LBP, leading to more targeted and mechanism-based treatments.^[21,22]^ Therefore, the aim of this study is to explore which lifestyle and psychological factors are associated with altered central pain processing in service members with persistent LBP.

## 2. Methods

### 2.1. Study design

This is a cross-sectional exploratory observational study. The study was approved by the scientific and ethical review board of Vrije Universiteit Amsterdam (VCWE-2020-080) and the Medical Ethical Committee Zuyderland-Zuyd (METCZ220200063). All participants signed written informed consent prior to participation. The study was performed according to the principles of the Declaration of Helsinki (2013) and reported according to the STROBE guidelines.^[31,32]^

### 2.2. Setting

Participants were recruited from the in- and outpatient population of Military Rehabilitation Centre (MRC) “Aardenburg,” Doorn, The Netherlands, between August 2020 and January 2021. The MRC receives service members referred by a military general practitioner or medical specialist. All participants were treated with a multidisciplinary approach.

### 2.3. Participants

Participants were eligible for participation if they: followed a rehabilitation program at the MRC for LBP, had LBP for at least 12 weeks, had LBP with an average severity of ≥ 3 on the Numeric Pain Rating Scale (NPRS; “0” indicating “no pain” and “10” indicating “most intense pain imaginable”) within the last 7 days, were employed in the Dutch armed forces, were at least 18 years of age and not older than 65 years, and had sufficient knowledge of the Dutch language to complete the questionnaires and understand the instructions. Participants were excluded if they had any serious spinal pathologies (radiculopathy, stenosis, spinal tumors or infections, vertebral fracture, osteoporosis, ankylosing spondylitis, spondylolisthesis or previous back surgery). Additionally, participants were requested not to take any pain medication, and not to consume any alcohol in the 24 hours prior to the measurements.

### 2.4. Quantitative sensory testing

#### 2.4.1. Procedure.

The procedure consisted of QST measurements i.e., TS and CPM, to assess central pain processing, and questionnaires regarding potential associated factors. The entire procedure took 90 minutes, was conducted between 10 a.m. and 3 p.m., and took place in a room with a constant temperature between 20°C and 22°C. The QST measurements were performed by a trained examiner (JMP). The questionnaires were self-reported, and the examiner was blinded for these outcomes. The procedure started with participants completing the self-reported questionnaires for 15 minutes, followed by the TS test. Subsequently there was a wash-out period of 15 minutes during which the participants continued completing the self-reported questionnaires. Lastly, the CPM test was performed. The order of the questionnaires was randomized by computer (https://commentpicker.com/nl/lijst-randomiseren.php) to prevent attentional bias.

During QST, participants were positioned comfortably lying face down on a physiotherapy plinth.^[33]^ Standard instructions were provided before the tests and the test locations were marked. Both tests were performed on 2 anatomical locations: on a local (painful) test side and on a remote test side.^[33]^ The local test side was paraspinal 2 cm lateral of the L4 spinous process on the erector spinae muscles, on the body side with the highest self-reported pain intensity.^[34]^ In case both body sides were equally painful, the side with (highest intensity of) radiating leg pain was used. If the participant reported no difference in radiating leg pain either, the body side was randomly selected.^[35]^ The remote test side was on the extensor carpi radialis longus muscle (5 cm distal to lateral epicondyle of humerus) on the same body side as the local location.^[36]^ The order of the testing locations (local or remote) was randomized by computer for the first measurement and this order was maintained, alternating the 2 locations, for the remaining measurements.^[37]^ Before the actual test, a familiarization measurement was conducted on the extensor carpi radialis longus muscle, on the body side that was not involved in the QST battery.^[33]^

### 2.5. Outcome measures

#### 2.5.1. Temporal summation of pain.

First, a single stimulus was applied on the skin using a 256-mN PinPrick stimulator (MRC Systems, Heidelberg, Germany) and participants were asked to rate their pain intensity on a NPRS 0 to 10. After 30 seconds a train of 10 stimuli was applied at 1Hz within an area of 1 cm^2^. Participants also rated their pain intensity of the 10th stimulus. The whole procedure was performed 5 times per test location, with 30 seconds between the measurements. The mean NPRS score of the single stimulus was subtracted from the mean NPRS score of the 10th stimulus of the trains, representing the absolute TS effect.^[38]^ In addition, the wind-up ratio (WUR) was calculated by dividing the mean NPRS score of the 10th stimulus of the series, by the mean NPRS score of the single stimulus.^[39]^ This procedure was according to the standardized protocol of the German Research Network on Neuropathic Pain.^[39]^

### 2.6. Conditioned pain modulation

#### 2.6.1. Test stimulus.

First, the test stimulus was conducted as baseline measurement (PPT1). The test stimulus was the mechanical pressure pain thresholds (PPT) test according to the QST protocol of the German Research Network.^[39]^ A digital algometer (Type II, Somedic AB, Stockholm, Sweden) with a 1 cm^2^ probe, was used to progressively apply pressure with an application rate of 50 kPa/s. At the moment the pressure sensation changed to a painful sensation, the participant pressed the hand-held switch and the algometer was released.^[33]^ The amount of pressure (kPa) at which the sensation of pressure transitioned to the sensation of pain, was recorded by the algometer. The procedure was performed twice per test location, with an interstimulus time of 20 seconds between the measurements. The reported PPT was the mean pressure intensity of the 2 measurements.

#### 2.6.2. Conditioning stimulus.

A parallel paradigm was used to measure CPM, meaning the conditioning stimulus was applied before and during the test stimulus (PPT2).^[34,40]^ The conditioning stimulus was the cold pressor test by submerging the hand, with spread and lightly moving fingers (contralateral body side of the PPT measurement), up to the wrist in a cold water bath of 8 to 9°C.^[41]^ Participants were instructed to keep their hand in the cold water bath until the test stimulus (PPT2), which started 60 seconds after immersing the hand, was completed or the pain became intolerable.^[34,40]^ The absolute CPM-effect was calculated by subtracting the mean pressure intensity of the baseline PPT (PPT1) from the mean pressure intensity of the test stimulus (PPT2). In addition, the relative CPM-effect was calculated by the following formula: ((PPT2/PPT1)-1) × 100. For both the absolute and relative CPM-effect, a positive value represented pain inhibition.^[42]^

### 2.7. Demographic and clinical characteristics

Data related to age, sex, duration of LBP, smoking behavior, diagnosis of post-traumatic stress disorder, military service and rank were collected through a self-reported questionnaire. In addition, the average LBP intensity of the past 7 days was obtained using an 11-point NPRS. The NPRS is considered valid for the use in chronic pain.^[43]^ The perceived LBP-associated disability was measured using the Roland Morris Disability Questionnaire (RMDQ). The RMDQ is a reliable and valid questionnaire consisting of 24 items referring to different activities that may be affected by LBP. Participants were asked to select the items which described the disabilities they experienced.^[44]^

### 2.8. Candidate associated factors

The selection of candidate associated factors was based on previous studies assessing factors associated with altered central pain processing^[26,27,34]^ and clinical relevance for the military population.^[45]^ The selected candidate associated factors and measurement tools are described below.

#### 2.8.1. Alcohol use.

The Alcohol Use Disorders Identification Test was used to identify excessive drinking. It is a 10-item questionnaire which covers the domains of alcohol consumption, alcohol dependence and alcohol-related problems. The total score ranges from 0 to 40 (higher scores indicating more at-risk drinking).^[46]^ The Alcohol Use Disorders Identification Test was found to be valid and reliable.^[47]^

#### 2.8.2. Body mass index.

As recommended by the World Health Organization, body mass index (BMI) was used as a measure for relative bodyweight.^[48]^ BMI is calculated by dividing the self-reported bodyweight in kilograms by the square of the body height in meters (kg/m^2^).

#### 2.8.3. Physical activity and sitting time.

The long version of the International Physical Activity Questionnaires (IPAQ) was used to collect data on the level of physical activity. The IPAQ consists of 31 items about household and yard work activities, occupational activity, self-powered transport, and leisure-time physical activity as well as sedentary activity over the last 7 days. For all activities, the metabolic equivalent of task (MET)-minutes per week (MET·min·wk^−1^) was calculated by multiplying the MET value by the duration in minutes and multiplying this by the frequency per week. The MET-minutes per week were summed for all activities to obtain the total amount of physical activity over the last week. In addition, the total minutes sitting activities per week was calculated. The IPAQ is a reliable and valid questionnaire for collecting physical activity data.^[49]^

#### 2.8.4. Pain catastrophizing.

The Pain Catastrophic Scale (PCS) is a validated 13-item questionnaire which measures thoughts and feelings related to pain, suggestive of catastrophic cognitions. The PCS consists of the 3 subscales magnification, rumination and helplessness. Participants scored to what extent they experienced these catastrophic thoughts on a 5-point scale (0 = not at all, 4 = all the time). Total scores range from 0 to 52.^[50]^

#### 2.8.5. Symptoms of anxiety and depression, and sleep quality.

The Symptom Checklist-90-Revised (SCL-90-R) is a validated questionnaire in which participants note on a 5-point scale (1 = totally not, 5 = very much) to what extent they experienced various physical and psychological symptoms of distress in the last 7 days.^[51]^ In the present study, the subscales: anxiety, depression and sleep difficulties of the Dutch version of the SCL-90-R were used.^[52]^

### 2.9. Sample size

A sample size calculation was performed using G*Power, version 3.1.9. Due to the explorative character of the study, statistical significance was set on 0.10. To obtain an effect size of f2 = 0.5 and a power of 0.80 in a model with 3 factors, twenty-one participants were required.

### 2.10. Statistical analysis

Descriptive statistics were reported as means with standard deviations (SD), medians with interquartile ranges (IQR) or absolute numbers (n) with percentages. Data were assessed for missing values. Furthermore, if the pain rating of the single stimulus was 0/10 in more than 3/5 TS measurements, the WUR could not be calculated and the case was excluded for WUR.^[53]^ If this occurred in ≥ 30% of the subjects, the WUR and absolute TS were excluded for further analysis.

Univariable regression analyses were performed after assessing the assumption of normality by visual inspection of the Q-Q plot, histogram and box plot, and performing a Shapiro–Wilk test. Moreover, the residuals were visually assessed for normal distribution and homoscedasticity. If one of the assumptions was violated, bootstrapping of 1000 samples was used to generate bias-corrected and accelerated (BCa) 90% confidence intervals and significance tests.^[54]^ The dependent variables of the regression analyses included all 8 QST outcomes i.e., absolute TS, WUR, absolute CPM and relative CPM on both the local and remote test side. The independent variables were the aforementioned candidate associated factors. Unstandardized beta coefficients (Unstandardized B) with 90% confidence intervals (90% CI), standardized beta coefficients (Standardized β), and significance levels (*P* value) were reported. *P* values of less than .1 were considered significant due to the exploratory nature of the study.

In addition to the univariable regression analyses, multivariable regression analyses were performed to adjust for potential confounding and/or effect modification through age.^[26,55]^ To assess confounding effects, age was added to the model and if the unstandardized coefficient changed with > 10%, this adjusted model was accepted. To assess effect modification, an interaction term was added to the model, and if the interaction was significant, this adjusted model was reported. Statistical analysis was conducted with SPSS version 28 (IBM NY SPSS Statistics, IBM Corp., Armonk, NY).

## 3. Results

### 3.1. Participants

**Figure 1.**
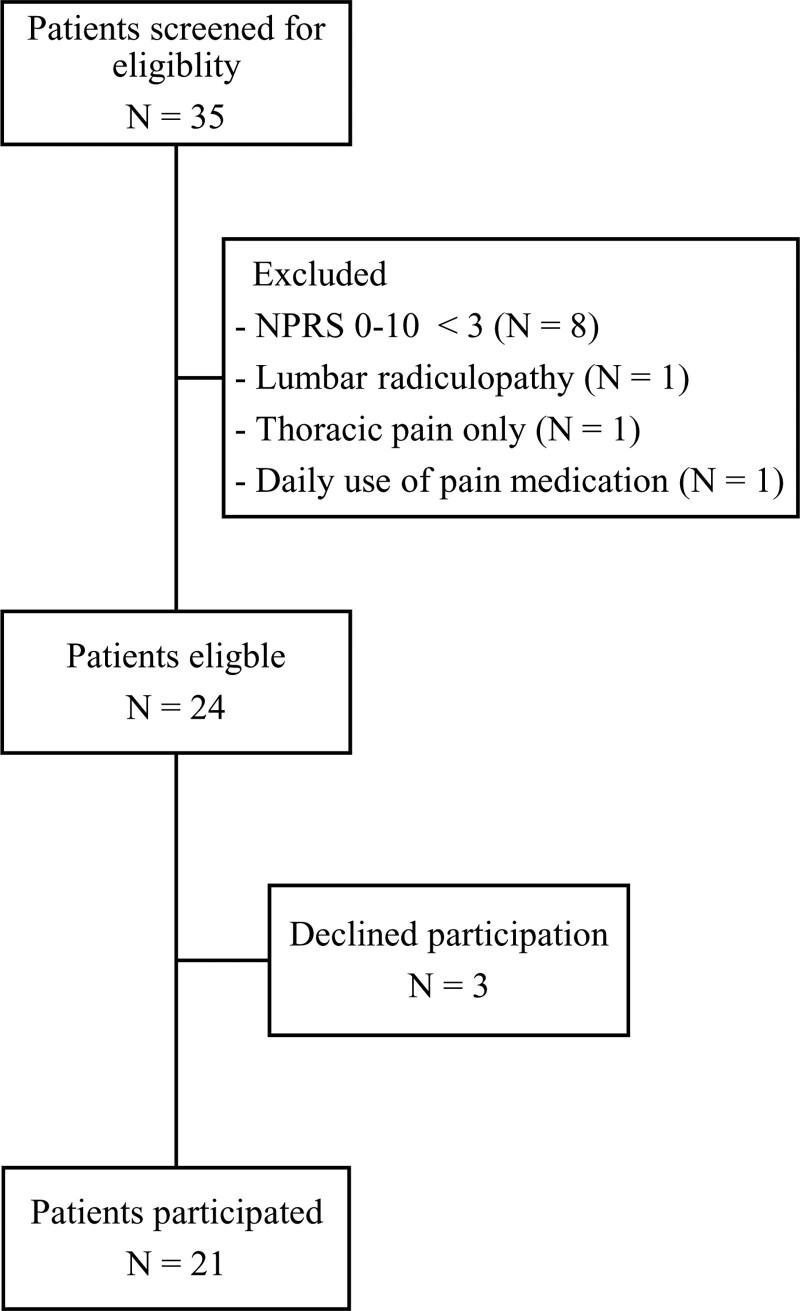
Flowchart inclusion process. NPRS = numeric pain rating scale.

Thirty-five potential eligible participants were approached and screened by the researcher. Twenty-four participants were eligible for inclusion of which twenty-one agreed to participate in the study and signed written informed consent (Fig. 1).

### 3.2. Descriptive data

Demographic and clinical characteristics, and outcomes of the questionnaires are presented in Table 1. In the IPAQ questionnaire, results of one participant (4.8%) were missing. This small amount was considered acceptable.^[56]^ We therefore waived a missing value imputation procedure.

**Table 1:** Demographic characteristics and questionnaire outcomes (N = 21).

Characteristic	
Age (years) mean (SD)	34.0 (9.5)
Sex n (%)	
Male	18 (85.7)
Female	3 (14.3)
BMI (kg/m^2^) mean (SD)	26.1 (2.2)
Duration of PLBP (weeks) median (IQR)	156.0 (67.5–280.0)
NPRS (0–10) median (IQR)	4.0 (3.0–5.0)
RMDQ (0–24) mean (SD)	7.0 (4.5)
Smoking n (%)	
Yes	5 (23.8%)
No	16 (76.2%)
PTSD n (%)	
Yes	1 (4.8)
No	20 (95.2)
Service n (%)	
Royal Netherlands Army	11 (52.4)
Royal Netherlands Navy	4 (19.0)
Royal Netherlands Air Force	1 (4.8)
Royal Netherlands Military Police	5 (23.8)
Rank n (%)	
Enlisted personnel	7 (33.3)
Noncommissioned officers	10 (47.6)
Commissioned officers	4 (19.0)
AUDIT (0–40) mean (SD)	6.5 (2.7)
IPAQ median (IQR)	
Physical activity (MET/min/wk)	4852.0 (3258.4–8030.3)
Sitting (min/wk)	2220.0 (1515.0–3480.0)
PCS (0–52) mean (SD)	16.4 (8.0)
SCL-90-R median (IQR)	
Anxiety (10–50)	12.0 (10.5–15.5)
Depression (16–80)	20.0 (17.5–23.0)
Sleep (3–15)	5.0 (3.0–6.5)

AUDIT = alcohol use disorders identification test, BMI = body mass index, IPAQ = international physical activity questionnaire, IQR = interquartile range, NPRS = numeric pain rating scale, PCS = pain catastrophic scale, PLBP = persistent low-back pain, PTSD = post-traumatic stress disorder, RMDQ = Roland Morris disability questionnaire, SCL-90-R = symptom checklist-90-revised, SD = standard deviation.

Table 2 shows the mean results of the QST measures. For the single measures of TS on the local test side, 5 subjects scored 0 on the NPRS (0–10) in > 3/5 measurements, therefore the WUR scores could not be calculated in these participants. On the remote test side, 10 subjects scored the single TS measure as 0 on the NPRS (0–10) in > 3/5 measurements, hence the absolute TS and WUR on the remote test side were excluded from further analysis. The absolute CPM effect (~95 k/Pa) was comparable at the local and remote site.

**Table 2:** Mean results quantitative sensory testing (N = 21).

QST measure	Local test side mean (SD)	Remote test side mean (SD)
Absolute TS	1.9 (1.4)	–
WUR*	2.7 (1.6)	–
Absolute CPM	94.6 (139.4)	96.7 (107.6)
Relative CPM	16.4% (24.7)	29.6% (38.1)

Positive CPM values represent pain inhibition.

Local test side: paraspinal 2 cm lateral of the L4 spinous process on the erector spinae muscles.

Remote test side: on the extensor carpi radialis longus muscle (5 cm distal to lateral epicondyle of humerus).

CPM = conditioned pain modulation, QST = quantitative sensory testing, SD = standard deviation, TS = temporal summation of pain, WUR = wind-up ratio.

* For WUR: N=16.

### 3.3. Factors associated with temporal summation of pain

**Figure 2.**
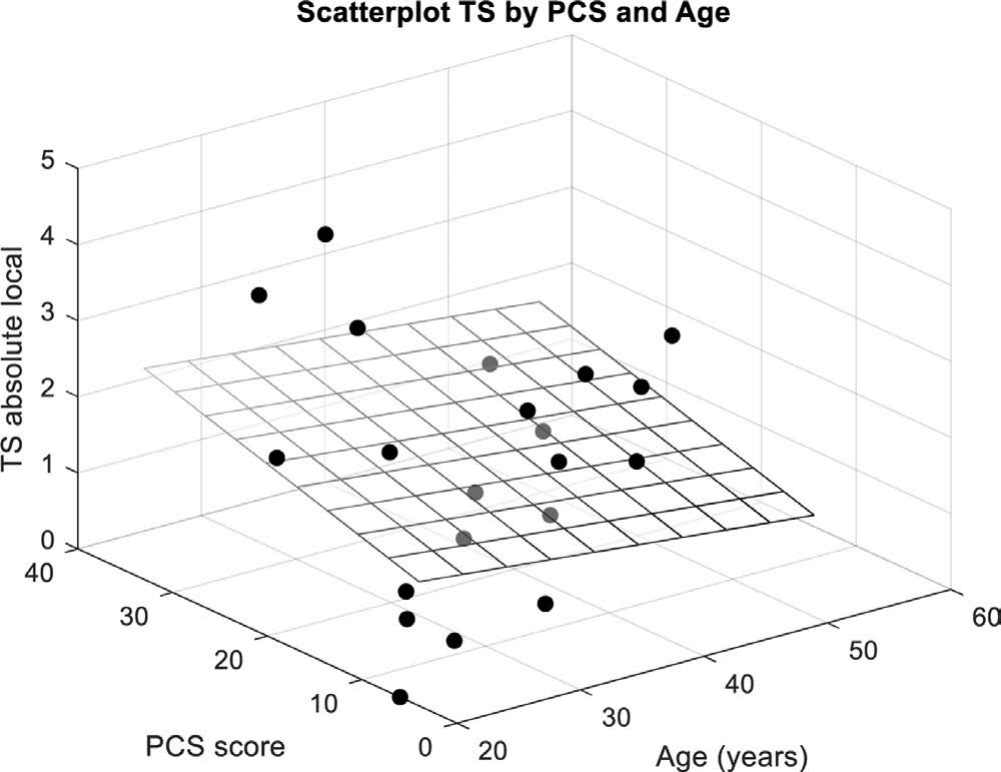
Scatterplot for the association between absolute local temporal summation and pain catastrophizing corrected for the effect modifying effect of age. PCS = pain catastrophic scale, TS = temporal summation.

**Figure 3.**
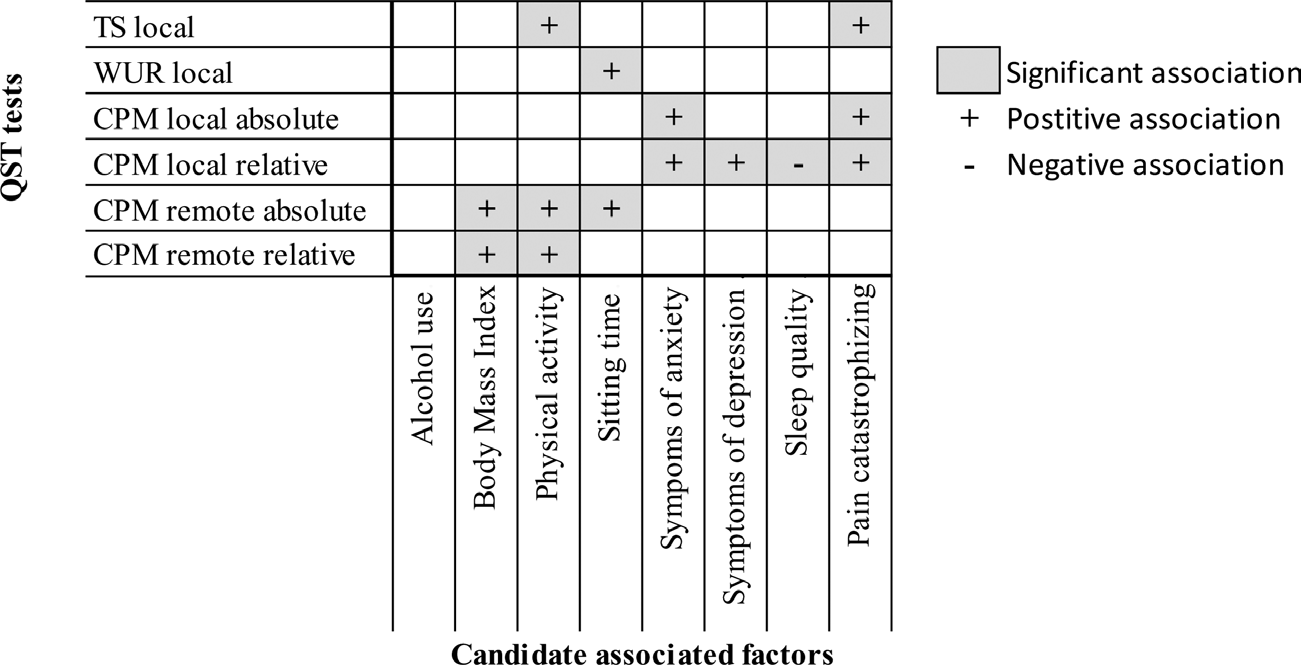
Overview of (non)significant associations. CPM = conditioned pain modulation, TS = temporal summation, QST = quantitative sensory testing, WUR = wind-up ratio. If available, adjusted models were used in this figure.

A higher level of physical activity was significantly associated with a higher TS, and a longer sitting time was significantly associated with a higher WUR (Table 3). In addition, after correcting for age as effect modifier, a higher level pain catastrophizing was significantly associated with a higher TS (Fig. 2). The other factors did not show a significant univariable or multivariable association with TS. Figure 3 provides an overview of the (non)significant associations.

**Table 3:** Regression analysis temporal summation of pain and wind-up ratio.

Variables	Model	TS local			WUR local		
		Unstandardized B (90% CI)	Standardized β	*P* value	Unstandardized B (90% CI)	Standardized β	*P* value
Alcohol use (AUDIT)	Crude	−0.039 (−0.236 to 0.158)	−0.078	.738	0.117 (−0.140 to 0.375)	0.210	.435
BMI (kg/m^2)^	Crude	−0.109 (−0.355 to 0.137)	−0.174	.452	0.201 (−0.066 to 0.471)‡	0.247	.238‡
Physical activity (IPAQ)	Crude	0.000 (0.000–0.000)	0.551	**.012**	3.774E-5 (0.000 to 0.000)‡	0.117	.584‡
Sitting time (IPAQ)	Crude	0.000 (0.000–0.001)	0.171	.472	0.001 (0.000–0.001)	0.532	**.034**
Anxiety (SCL-90-R)	Crude	−0.034 (−0.148 to 0.081)	−0.116	.615	−0.046 (−0.207 to 0.116)	−0.132	.626
	Adjusted				−0.061 (−0.234 to 0.112)*	−0.177*	.544*
Depression (SCL-90-R)	Crude	−0.014 (−0.119 to 0.091)	−0.052	.117	−0.065 (−0.302 to 0.171)	−0.129	.634
	Adjusted	−0.012 (−0.125 to 0.101)*	−0.044*	.860*	−0.094 (−0.352 to 0.165)*	−0.185*	.532*
Sleep (SCL-90-R)	Crude	0.000 (−0.201 to 0.201)	0.000	1.000	−0.135 (−0.587 to 0.316)	−0.139	.606
	Adjusted				2.710 (−0.176 to 5.596)†	2.798†	.120†
Pain catastrophizing (PCS)	Crude	0.033 (−0.034 to 0.100)	0.191	.406	0.013 (−0.101 to 0.128)	0.055	.839
	Adjusted	0.349 (0.107–0.591)†	2.027†	**.023** †	0.006 (−0.118 to 0.131)*	0.026*	.929*

Bold values denote statistical significance at the *P* < 0.1 level.

AUDIT = Alcohol Use Disorders Identification Test, BMI = body mass index, IPAQ = International Physical Activity Questionnaire, PCS = pain catastrophic scale, SCL-90-R = Symptom Checklist-90-Revised.

* Adjusted for the confounding effect of age.

† Adjusted for the effect modifying effect of age.

‡ Confidence intervals and significance tests are bias-corrected and accelerated based on bootstrapping of 1000 samples.

### 3.4. Factors associated with conditioned pain modulation

**Figure 4.**
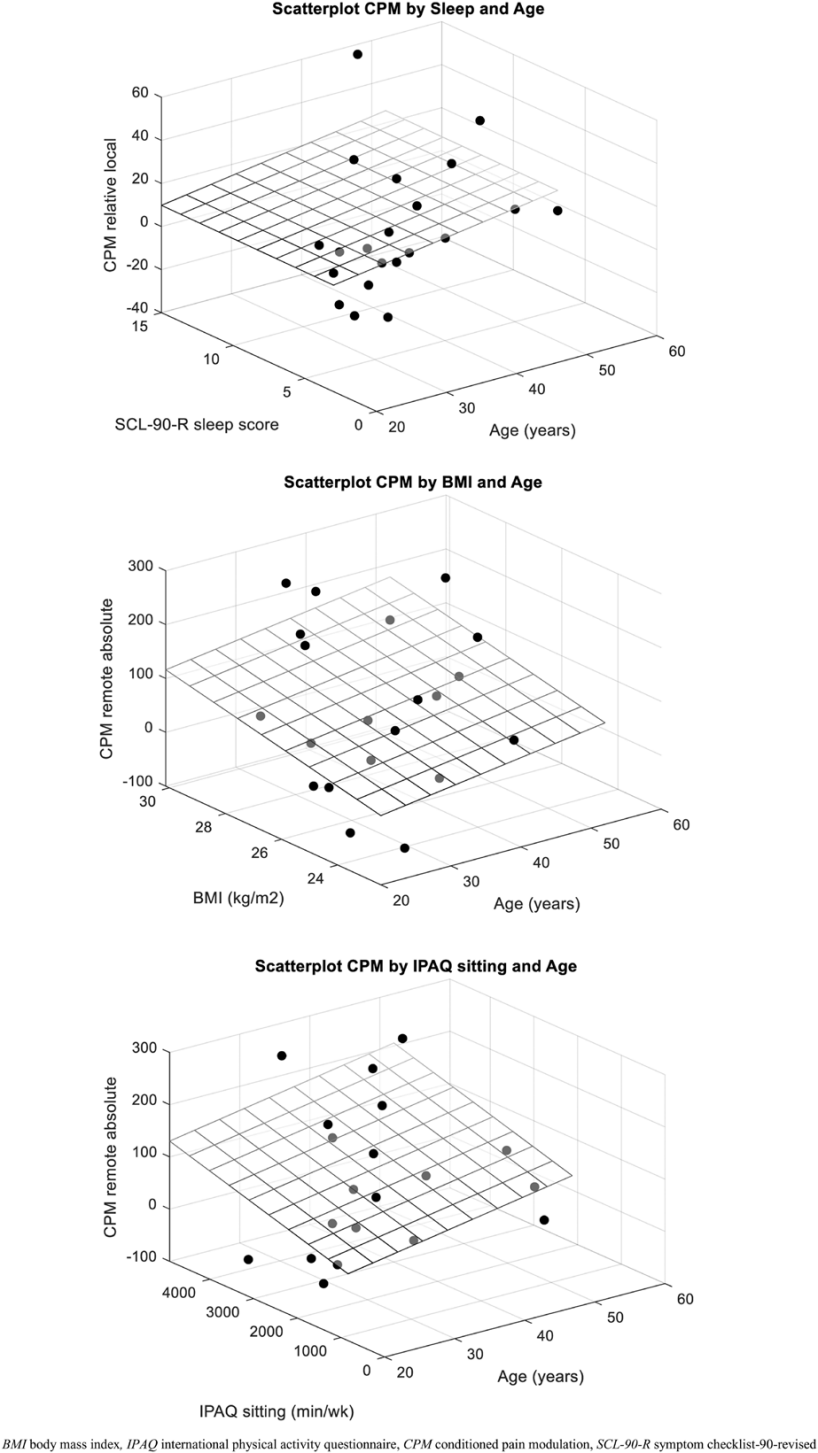
Scatterplots for the associations between relative local conditioned pain modulation and sleep difficulties, absolute remote conditioned pain modulation and body mass index, and absolute remote conditioned pain modulation and sitting time, all corrected for the effect modifying effect of age. BMI = body mass index, CPM = conditioned pain modulation, IPAQ = international physical activity questionnaire, SCL-90-R = symptom checklist-90-revised.

A higher local absolute CPM was significantly associated with a higher level of pain catastrophizing and a higher degree of anxiety symptoms (Fig. 3). These factors were also significantly associated with a higher local relative CPM (Table 4). In addition, a higher local relative CPM was also associated with a higher degree of depression symptoms and, after correcting for age as an effect modifier, with less sleep difficulties (Fig. 4).

**Table 4:** Regression analysis conditioned pain modulation local.

Variables	Model	CPM local absolute			CPM local relative		
		Unstandardized B (90% CI)	Standardized β	*P* value	Unstandardized B (90% CI)	Standardized β	*P* value
Alcohol use (AUDIT)	Crude	−5.007 (−25.013 to 15.000)	−0.099	.670	−0.032 (−3.594 to 3530)	−0.004	.988
	Adjusted	−4.194 (−24.452 to 16.065)*	−0.083*	.724*	0.107 (−3.505 to 3.719)*	0.012*	.960*
BMI (kg/m^2)^	Crude	−14.280 (−39.017 to 10.458)	−0.223	.331	−2.240 (−6.647 to 2.168)	−0.198	.391
	Adjusted	−23.278 (−49.507 to 2.951)*	−0.364*	.141*	−3.704 (−8.425 to 1.017)*	−0.327*	.190*
Physical activity (IPAQ)	Crude	0.005 (−0.005 to 0.015)	0.185	.436	0.001 (−0.001 to 0.003)	0.200	.397
	Adjusted	0.007 (−0.004 to 0.017)*	0.261*	.290*			
Sitting time (IPAQ)	Crude	−0.008 (−0.060 to 0.044)	−0.064	.789	−0.002 (−0.011 to 0.007)	−0.083	.729
	Adjusted	−0.012 (−0.065 to 0.041)*	−0.094*	.700*			
Anxiety (SCL-90-R)	Crude	14.144 (3.857 to 24.430)	0.479	**.028**	2.256 (0.382 to 4.129)	0.431	**.051**
Depression (SCL-90-R)	Crude	9.172 (−0.861 to 19.206)	0.341	.130	1.805 (0.055 to 3.555)	0.379	**.090**
	Adjusted	8.245 (−2.508 to 18.998)*	0.306*	.200*			
Sleep (SCL-90-R)	Crude	1.057 (−19.354 to 21.468)	0.021	.930	0.758 (−2.846 to 4.363)	0.083	.720
	Adjusted	−4.052 (−26.601 to 8.497)*	−0.079*	.759*	−21.554 (−40.488 to −2.620)†	−2.364†	**.064** †
Pain catastrophizing (PCS)	Crude	8.214 (2.079–14.348)	0.469	**.032**	1.500 (0.423–2.577)	0.484	**.026**

Bold values denote statistical significance at the *P* < 0.1 level.

AUDIT = alcohol use disorders identification test, BMI = body mass index, IPAQ = international physical activity questionnaire, PCS = pain catastrophic scale, SCL-90-R = symptom checklist-90-revised.

* Adjusted for the confounding effect of age.

† Adjusted for the effect modifying effect of age.

After correcting for age as confounder, a higher remote absolute CPM was significantly associated with a higher level of physical activity. In addition, a higher BMI and lower sitting time were significantly associated with a higher remote CPM effect after correcting for age as effect modifier (Table 5; Fig. 4). A higher BMI and a higher level of physical activity were, corrected for the confounding effect of age, also associated with a higher remote relative CPM. None of the other candidate variables showed a significant association with CPM.

**Table 5:** Regression analysis conditioned pain modulation remote.

Variables	Model	CPM remote absolute			CPM remote relative		
		Unstandardized B (90% CI)	Standardized β	*P* value	Unstandardized B (90% CI)	Standardized β	*P* value
Alcohol use (AUDIT)	Crude	−0.591 (−16.107 to 14.925)	−0.015	.948	−2.318 (−7.729 to 3.094)	−0.168	.468
	Adjusted	0.219 (−15.277 to 15.715)*	0.006*	.981*	−2.064 (−7.505 to 3.377)*	−0.149*	.519*
BMI (kg/m^2)^	Crude	14.686 (−4.016 to 33.387)	0.297	.190	2.643 (−4.206 to 9.492)	0.151	.531
	Adjusted	87.046 (15.261 to 158.831)†	1.763†	**.050** †	29.447 (3.519 to 55.374)†	1.686†	**.065** †
Physical activity (IPAQ)	Crude	0.007 (0.000 to 0.014)	0.380	**.098**	0.003 (0.001–0.006)	0.469	**.037**
	Adjusted	0.009 (0.002 to 0.016)*	0.488*	**.036** *	0.004 (0.002–0.006)*	0.580*	**.011** *
Sitting time (IPAQ)	Crude	0.028 (−0.010 to 0.66)	0.287	.220	0.008 (−0.006 to −0.022)	0.238	.312
	Adjusted	−0.210 (−0.406 to −0.015)†	−2.156†	**.079** †	0.007 (−0.007 to 0.021)*	0.210*	.380*
Anxiety (SCL-90-R)	Crude	1.684 (−7.335 to 10.704)	0.074	.750	0.069 (−3.130 to 3.268)	0.009	.970
	Adjusted	0.743 (−8.377 to 9.863)*	0.033*	.889*	−0.255 (−3.496 to 2.986)*	−0.032*	.893*
Depression (SCL-90-R)	Crude	−3.533 (−11.651 to 4.584)	−0.170	.461	−1.323 (−4.189 to 1.543)	−0.180	.435
	Adjusted	−5.626 (−13.901 to 2.649)*	−0.271*	.254*	−2.030 (−4.967 to 0.907)*	−0.276*	.246*
Sleep (SCL-90-R)	Crude	−7.259 (−22.751 to 8.233)	−0.183	.428	−1.659 (−7.194 to 3.876)	−0.118	.610
	Adjusted	−13.890 (−30.161 to 2.380)*	−0.350*	.156*	−3.697 (−9.631 to 2.237)*	−0.263*	.294*
Pain catastrophizing (PCS)	Crude	−1.029 (−6.375 to 4.317)	−0.76	.743	−0.434 (−2.323 to 1.455)	−0.091	.695
	Adjusted	−2.432 (−7.991 to 3.128)*	−0.180*	.458*	−0.909 (−2.881 to 1.063)*	−0.190*	.435*

Bold values denote statistical significance at the *P* < 0.1 level.

AUDIT = alcohol use disorders identification test, BMI = body mass index, IPAQ = international physical activity questionnaire, PCS = pain catastrophic scale, *SCL-90-R* = symptom checklist-90-revised.

* Adjusted for the confounding effect of age.

† Adjusted for the effect modifying effect of age.

## 4. Discussion

The aim of this study in service members with persistent LBP was to explore which lifestyle and psychological factors were associated with altered central pain processing. Our main findings were that in service members with persistent LBP, a higher pain facilitatory function, measured by TS on the lower back, was significantly associated with a higher self-reported level of physical activity, a longer sitting time and a higher level of pain catastrophizing. Less effective pain inhibition, measured by CPM on the lower back, was significantly associated with a lower level of pain catastrophizing, a lower degree of depression and anxiety symptoms and a higher degree of sleep difficulties. A lower CPM measured on the forearm was significantly associated with a lower BMI, a lower level of physical activity and a longer sitting time.

### 4.1. Temporal summation of pain

Positive associations were found between WUR and sitting time and the level of physical activity. Two earlier studies have also assessed these relationships and found a lower TS to be associated with higher levels of self-reported vigorous and total physical activity in healthy individuals and with objectively measured moderate vigorous physical activity in older adults.^[57,58]^ The results regarding the level of physical activity in the present study, contrast the results of these earlier studies. This may, in part, be explained by the occupational physical activity in the military, which includes carrying heavy loads and wearing heavy armor.^[59]^ Almost all participants in the present study (18/21) had jobs with heavy physical demands, hence we were unable to statistically correct for this factor. Notwithstanding, heavy physical tasks, as performed by most of the participants, account for a substantial amount of acute low-back injuries, i.e. may cause nociceptive input in the lower back.^[59,60]^ Nociceptive input is considered to initiate and/or maintain altered pain processing.^[13,61]^ Therefore, it can be argued that the distinctive nature of physical activity in the military, accounts for the enhanced pain facilitation associated with high levels of physical activity, thereby distinguishing service members from the civilian population.

Pain catastrophizing, was found to be positively associated with TS. This finding is in line with previous research in persistent LBP patients.^[62,63]^ The relationship is further supported by brain research, showing an increased brain activity in areas related to anticipation of pain, attention to pain and emotional aspects of pain in individuals with higher levels of pain catastrophizing.^[64]^

### 4.2. Conditioned pain modulation

Multiple factors associated with CPM were also identified. First of all, poor sleep was found to be related to a lower local CPM. These findings are in accordance with a previous study in acute LBP patients.^[34]^ It has been suggested that the relationship between sleep and pain is bidirectional: pain may cause sleep deprivation and poor sleep may again affect pain.^[34]^ The effect of sleep disturbances on pain has been attributed to aberrant glial activity, which is considered an underlying mechanism of central sensitization.^[65]^ Secondly, a positive association between BMI and remote CPM was found. This association seems surprising as overweight is considered a risk factor for LBP.^[66]^ However, BMI is a measure of relative bodyweight rather than body composition.^[67]^ Therefore, a high BMI may reflect high levels of skeletal muscle mass, rather than fat mass, in individuals with well-developed musculature like service members.^[68]^ Skeletal muscle mass was previously found to increase the CPM effect, likely explaining the results of the current study.^[69]^ Finally, a higher level of physical activity was related to a higher remote CPM. This relationship seems paradoxical, considering a higher level of physical activity was also related to a higher TS. This implies that physical activity induces 2 concurrent processes with opposing effects. The effect of physical activity on TS may, as we hypothesized before, result from ongoing nociceptive input, whereas the higher CPM is likely induced by an activation of opioids and dopamine.^[70]^

Higher levels of pain catastrophizing and symptoms of anxiety and depression were associated with a greater local CPM. These results regarding psychological factors seem counterintuitive, considering symptoms of depression and anxiety are recognized predictors for chronicity in low-back pain, and psychological processes and CPM largely share the same neurotransmitters.^[27,71]^ However, this apparent relationship was also absent in a meta-analysis^[27]^ and a large recent study,^[72]^ both of which reported no significant associations between psychological factors and CPM. The positive associations observed in the current study, as opposed to the absence of such association in those previous studies, can be explained by 2 possible reasons. First, the considerably more homogeneous population in the current study, in contrast to the more diverse populations in the previous mentioned studies,^[27,72]^ substantially increased the likelihood of finding significant associations. Secondly, the influence of the pressure-based CPM paradigm could explain our results, as the meta-analysis mentioned earlier did not find any links between CPM and psychological factors when considering all modalities collectively, yet it revealed a correlation between pressure-based CPM and anxiety.^[27]^ This was explained by the dual impact of deep pressure, which not only provokes pain but also acts as a therapeutic method to induce calmness and reduce anxiety. It is possible, that the use of a pressure-based paradigm accounts for the results of the current study, and using a different test-stimulus modality might have yielded different results.

### 4.3. Generalizability

Only 3 females were included in the current study. Nevertheless, similar percentages of females were reported in a larger study in service members, hence the sex distribution is considered representative.^[45]^ Yet, numerous patients were excluded for having a NPRS-10 score of less than 3. Consequently, this study does not reflect the population of service members with low intensities of LBP. It should also be noted that although only patients with a NPRS-10 score of at least a score of 3 out of 10 were included, the median pain intensity of 4 was still low compared with similar studies in civilian populations.^[34,73]^ This difference might be attributed to the high physical demands in the military. It is possible that civilians are still able to work with a NPRS score of 4, whereas service members cannot longer meet the high physical demands and are limited in performing their duties, forcing them to seek medical help.^[74,75]^

Also differences regarding pain processing results were found between the current study and studies in civilian populations with LBP.^[40,76]^ Two previous studies measured CPM in persistent LBP patients, also using the PPT at the lower back as test stimulus and the cold pressor test as conditioning stimulus, and reported notably lower mean CPM scores than found in the present study.^[40,76]^ This difference might be due to the majority of the participants of this study being male, considering males seem to have a more efficient CPM compared to females.^[26,77]^ Mean results regarding TS on the other hand, were comparable with studies in civilian populations with persistent LBP.^[78,79]^ Finally, the results regarding physical activity and BMI also indicate that findings in service members may differ significantly from findings in civilian populations. This underlines the disallowance of generalizing the results of the present study to civilian populations. With some caution, an exception can potentially be made for persistent LBP patients with similar occupational demands, like firefighters.

### 4.4. Strengths and limitations

A major strength of the present study is the limited sources of measurement bias, through controlling for room temperature, daypart of the measurements, daily use of pain medication and the use of painkillers, and alcohol consumption prior to the measurements. In addition, the examiner was blinded to the outcomes of the questionnaires and the order of the questionnaires was randomized. Nonetheless, the following limitations need to be addressed. Numerous participants scored the single pinprick stimulus as not painful. As a result, analysis of WUR on the local test side could only be performed on sixteen participants. On the remote test side, 10 participants scored the single stimulus as not painful, hence the whole variable was excluded. This problem could possibly have been avoided by using a 512-mN instead of a 256-mN PinPrick stimulator. On the other hand, a stronger PinPrick stimulator might have been too sensitive. A previous study in patients with sensory disturbances, reported that measurements had to be terminated because very sensitive patients experienced the stimulus of even the 256-mN PinPrick stimulator as too painful to continue.^[53]^ Another option may be to personalize the stimulus for each participant and/or use another instrument. For example by applying a stimulus with the amount of pressure needed to reach their PPT,^[80]^ or by administering heat at a temperature which the participant experiences as a pain score of 25/100.^[81]^ Another limitation is that factors associated with CPM might have been missed because only a pressure-based paradigm was used, considering certain factors were previously found to be associated with modality-specific CPM-effects.^[27]^ Experts recommended to use more than one test-stimulus,^[42]^ however to limit the burden on the participants, it was decided to only include one test stimulus. A final limitation is the cross-sectional nature of this study, which hinders the possibility to establish causality regarding the identified associations.

### 4.5. Recommendations and future studies

Important factors associated with altered central pain processing in service members with persistent LBP were identified and should be considered in future studies. To gain more insight in the relationships between the associated factors, future studies may want to augment the measurements of these factors. For example by, measuring body composition to further explain the results regarding BMI and collecting data on the amount of load carriage and wearing of heavy armor to examine the hypothesis that this may induce a higher TS. Additionally, prospective studies are needed to determine causality in these relationships. Finally, if future studies succeed in confirming and elaborating these results, treatments targeting these factors can be developed and expectedly improve pain processing and consequently the experienced pain in service members with persistent LBP.

## 5. Conclusion

In conclusion, our study demonstrates that level of physical activity, sitting time and pain catastrophizing are associated with TS. CPM was found to be related to BMI, level of physical activity, sitting time, sleep difficulties, symptoms of anxiety and depression, and pain catastrophizing. Though prospective studies are highly needed, this is an important first step towards identifying factors contributing to altered pain modulation in service members with persistent LBP.

## Acknowledgments

The authors thank the MSG Science Network Physiotherapy (https://www.msg-sciencenetwerk.nl/) for providing the measurement equipment, the Military Rehabilitation Centre “Aardenburg” for their support with the inclusion process and the service members for participation in the study.

## Author contributions

**Conceptualization:** Julia M. Prent, Peter van der Wurff, Gwendolyne G.M. Scholten-Peeters.

**Data curation:** Julia M. Prent.

**Formal analysis:** Julia M. Prent, Gwendolyne G.M. Scholten-Peeters.

**Investigation:** Julia M. Prent, Peter van der Wurff, Gwendolyne G.M. Scholten-Peeters.

**Methodology:** Julia M. Prent, Peter van der Wurff, Gwendolyne G.M. Scholten-Peeters.

**Project administration:** Julia M. Prent.

**Resources:** Peter van der Wurff, Gwendolyne G.M.Scholten-Peeters.

**Supervision:** Gwendolyne G.M. Scholten-Peeters.

**Visualization:** Julia M. Prent.

**Writing – original draft:** Julia M. Prent.

**Writing – review & editing:** Peter van der Wurff, Gwendolyne G.M. Scholten-Peeters.
